# Cyberbullying among Adolescents: Psychometric Properties of the CYB-AGS Cyber-Aggressor Scale

**DOI:** 10.3390/ijerph17093090

**Published:** 2020-04-29

**Authors:** Sofia Buelga, Javier Postigo, Belén Martínez-Ferrer, María-Jesús Cava, Jessica Ortega-Barón

**Affiliations:** 1 Department of Social Psychology, Faculty of Psychology, University of Valencia, Avda. Blasco Ibáñez, 21, 46010 Valencia, Spain; 2 Faculty of Psychology, University of Malaga, Bulevar Louis Pasteur, 25, 29010 Málaga, Spain; 3 Department of Education and Social Psychology, University Pablo Olavide, 41013 Sevilla, Spain; 4 Faculty of Education, International University of la Rioja (UNIR), Avenida de la Paz, 137, 26006 Logroño, Spain

**Keywords:** cyber-aggression, scale development, reliability, validity, adolescence

## Abstract

The present study aims to analyze the psychometric properties of the revised version of the Adolescent Cyber-Aggressor scale (CYB-AGS). This scale is composed of 18 items that measure direct and indirect cyberbullying. A cross-sectional study was conducted using two independent samples of adolescents. The first sample included 1318 adolescents (52.6% girls) from 12 to 16 years old (M = 13.89, SD = 1.32). The second sample included 1188 adolescents (48.5% boys) from 12 to 16 years old (M = 14.19, SD = 1.80). First, to study the psychometric properties of the CYB-AGS, exploratory factor analysis was performed on Sample 1. Results indicated a two-factor structure: direct cyber-aggression and indirect cyber-aggression. Second, to verify the structure of the CYB-AGS, we selected Sample 2 to conduct confirmatory factor analysis and test the scale’s convergent validity with theoretically-related measures. Results confirmed the reliability and validity of the two-dimensional model. Moreover, measurement invariance was established. Finally, regarding convergent validity, positive correlations were obtained between cyberbullying and aggressive behaviors in school, anger expression, negative attitudes towards school, and transgression of norms. Furthermore, negative correlations were found between cyberbullying and attitudes towards institutional authority.

## 1. Introduction

Fifth-generation mobile phone technologies (5G) are revolutionizing and transforming today’s digital era, creating an even more technological and interconnected society. The connection speed will be up to 100 times faster than the current one [1], and this change will make it possible to optimize an extensive environment of everyday objects connected to the Internet; Internet of Things (IoT) [2,3].

Currently, the smartphone is an indispensable everyday object in people’s lives, particularly in the daily lives of young people around the world [4,5,6]. In China, 90% of its 258 million young people have a smartphone they use to connect to their social networks daily [7]. In the United States, 95% of adolescents aged 13–17 own a smartphone and, of them, 45% report that they are online constantly [8]. This trend is also observed in Europe, especially in Spain, which is considered the European country with the largest number of smartphones in both the general population and among young people [9]. In fact, at age 12, 70% of Spanish adolescents have a smartphone, and 98% at age 14, which they often use to navigate without adult supervision [4,10].

In addition to the smartphone, a nearly universal device among adolescents of different genders, ethnicities, and socioeconomic backgrounds [8], the smartwatch is also beginning to play an increasingly important role in the lives of adolescents [9]. The popularity of this device is probably due to the fact that it is similar to a traditional watch (small and easy to carry at all times), but with the online communication features demanded by a generation that is “always on” [11,12].

New technologies certainly have important advantages, but they also have serious risks when they are misused [3], as in the case of cyberbullying. In this regard, it is very likely that 5G hyper-connectivity could increase the phenomenon of cyberbullying, which is already recognized as a major worldwide social and public health problem [5,13,14,15].

### 1.1. Cyberbullying in the “Always on” Smartphone Generation

Cyberbullying is defined as intentional, aggressive, and repetitive behavior, where a person or group uses electronic devices (mainly the Internet and the smartphone) to bully a person who cannot defend him/herself [5,16,17]. Anonymity, the 24/7 nature of the harassment, and a potentially large audience are specific characteristics of cyberbullying that foster and worsen the damage caused to the victim [18,19,20].

Online anonymity makes it easier for adolescents to engage in cyberbullying behavior towards others [21,22] because, unlike in traditional bullying, it is not necessary to be physically strong or belong to a powerful group in order to be a perpetrator. As Dennehy et al. [13] suggest, anyone can be a cyberbully online, motivated by internal factors, such as jealousy and revenge, and characteristics of the online environment that empower perpetrators. Although anonymity is never as invisible and secure as perpetrators believe, the difficulty of identifying them facilitates cyber-aggression perpetration at any time [17,21,22]. In Kowalski and Limber’s [23] study of over 3600 middle school students, almost 50% of the respondents did not know the identity of the individual who carried out the cyberbullying [24] (p. 21).

The helplessness and hopelessness the victim feels due to the perpetrator’s anonymity [25] is aggravated by the ease with which harmful content quickly goes viral [16,26] and the victim’s inability to stop it [24]. In this regard, some authors suggest that cyberbullying has more negative and adverse consequences for the victim than traditional bullying [27,28]. Of course, these detrimental effects are much more traumatic for the adolescent when he or she is subjected to a situation of poly-bullying victimization [29,30], which involves being a victim of both traditional bullying and cyberbullying [31,32,33] In fact, many authors show that there is a considerable overlap between traditional bullying and cyberbullying [4,24,25,34,35].

In addition, studies on the prevalence of cyberbullying among adolescents indicate that the incidence of this technological bullying ranges between 10% and 53% [35], with an average perpetration rate of 16% in the adolescent population [36,37]. These divergent rates across studies highlight the need for updated research tools that keep pace with society’s technological changes and show sufficient psychometric properties to measure new forms of cyberbullying with scientific rigor.

### 1.2. Measuring Cyberbullying

Continuous technological advances lead to new forms of cyberbullying that need to be identified and evaluated through scientific research in order to achieve their prevention and eradication. In recent years, considerable efforts have been made to validate new instruments that measure the types of cyberbullying behavior related to the different roles (victim, bully, and observer) involved in this type of violence. Thus, some authors have developed two-dimensional instruments that measure the role of the cyberbully and cyber-victim [38], whereas others have opted for a triangulated questionnaire that also includes the role of the bystander [39].

Although it is true that these instruments are interesting for the purposes of many studies, it is also important and necessary to have questionnaires that make it possible to analyze and closely examine a specific profile, such as the role of the cyber-victim [5] or the cyberbully [34,40]. For instance, in their study on cyberbullies, Wang et al. [7] conclude that the adverse effect of childhood maltreatment is related to adolescents’ cyberbullying perpetration. Moreover, Kabiri et al. [41] show the effects of life domains (self, family, school, and peers) on cyberbullying perpetration.

Thus, taking this background into consideration, and especially the current technological changes, a new version of the (CABS) scale was created and validated [42,43], in order to measure, as in the original scale, the role of the online aggressor. The CABS scale consisted of 10 items that measured harassment, insults, invasion of privacy, identity theft, and social exclusion [44] carried out by the adolescent in the past year. The new CYB-AGS scale adds eight more items related to new cyberbullying behaviors in order to evaluate two types of cyberbullying: direct and indirect [5,34,45].

Direct cyber-aggressions are behaviors and attacks directed at another person, both verbal (e.g., “I have sent someone taunting messages to bother and annoy him/her”) and social (e.g., “I have deleted or blocked someone from groups to leave him/her friendless”). Indirect cyber-aggressions are behaviors and attacks that use content manipulation (e.g., “I have created or manipulated videos or photos of someone”), identity theft (e.g., “I have pretended to be someone else to say or do bad things on the Internet or on social networks”), and hacking (e.g., “I have changed someone’s social network password so that he/she can’t access his/her accounts or social networks”: see Appendix A).

### 1.3. The Present Study

Therefore, the main objective of the present study is to validate the psychometric properties of the CYB-AGS scale. To do so, the structural validity of the instrument is analyzed by performing exploratory and confirmatory factorial analyses, multi-group analysis in two independent samples of adolescents, and analyses to test its convergent validity with indicators of psychosocial maladjustment. According to previous studies [5,34,45], we expect to find a two-factor model that groups cyber-aggressions into direct and indirect aggressions, with a good fit to the data. We also expect the scores on the CYB-AGS to correlate positively with aggressions at school, a negative attitude towards school, anger, and transgression of social norms, based on previous research indicating that these variables are related to cyberbullying [21,46].

## 2. Materials and Methods

### 2.1. Participants

The final sample consisted of 2506 adolescents enrolled in Obligatory Secondary Education. They were distributed in two independent samples, one from Andalucía and one from the Valencian Community (Spain). To select the participants, stratified cluster sampling was carried out. The sampling units were semi-public and public schools from the Valencian Community (Sample 1) and Andalusia (Sample 2). Sample 1 was composed of 1318 adolescents (52.6% girls; age range: 12–16 years, M = 13.89, SD = 1.32; 24.58% Grade 7, 27.39% Grade 8, 23.75% Grade 9, and 24.28% Grade 10). Sample 2 was composed of 1188 adolescents (48.5% boys; age range: 12–16 years, M = 14.19, SD = 1.80; 30% Grade 7, 25.10% Grade 8, 21.5% Grade 9, and 23.40% Grade 10). Prior analysis confirmed that the two samples were equivalent in terms of gender and academic grade.

### 2.2. Instruments

#### 2.2.1. Cyber-Aggression Scale (CYB-AGS)

The Cyber-aggression Scale (CYB-AGS) is an updated version of the Adolescent Aggression through Mobile Phone and Internet Scale (CYB-AG; [42]) that comprised 10 items. The new CYB-AGS scale adds eight more items related to new cyberbullying behaviors in order to asses two types of cyberbullying: direct and indirect. Hence, the CYB-AGS consists of 18 items (see Appendix A) rated on a 5–point Likert–type scale ranging from 1 (never) to 5 (always). These items measure the adolescent’s experience as a cyberbullying perpetrator in the past 12 months. Cronbach’s alpha for the global scale was acceptable (0.88).

Drawing on the items on the CYB-AG scale [42], and taking into account other scales that measure cyberbullying perpetration (e.g., [38], [47]), a list of 23 cyberbullying behaviors was elaborated. These changes were reviewed by a panel of six experts in cyberbullying in order to reach a consensus about the new items [48] and examine the items’ clarity and thoroughness. Next, the scale was administered to a pilot sample of 48 adolescents between 12 and 16 years old in order to explore possible difficulties with the items and find out whether the adolescents would add to or eliminate any cyberbullying behaviors from the scale. Some adolescents found one item from the list difficult to understand, and they thought that another item referred to a behavior that was not currently performed. At the same time, the expert panel coincided with the pilot sample and suggested suppressing another item. Thus, the scale was composed of 20 items. Then, two items with a saturation below 0.20 in the Exploratory Factor Analysis (EFA) were eliminated, and so the final scale contained 18 items.

#### 2.2.2. Assessment System for Children and Adolescents (Sistema de Evaluación de Niños y Adolescentes)

We selected two subscales from this questionnaire [49]. The first one, *negative attitudes towards school*, is composed of seven items, with scores ranging from 1 (never) to 5 (always), that assess negative attitudes towards studies and school (e.g., “I hate school”). The CFA using the maximum likelihood estimation method confirmed the fit of the proposed measurement model, SBχ2 = 50.74, df = 12, *p* < 0.001, CFI = 0.95, NNFI = 0.91, RMSEA = 0.07. Cronbach’s alpha coefficient for the present study was 0.81. The second subscale, scale of anger-control problems, contains eight items, rated on a scale ranging from 1 (never) to 5 (always), that assess aggressive behaviors due to difficulties in controlling anger (e.g., ‘When I am angry, I throw or break things’). The model showed an adequate fit to the data: SBχ2 = 63.61, df = 18, *p* < 0.001, CFI = 0.94, NNFI = 0.90, RMSEA = 0.06. Cronbach’s alpha coefficient in the present study was 0.79.

#### 2.2.3. Attitude towards Institutional Authority 

This two-factor scale [50] is composed of nine items, rated on a scale ranging from 1 to 4 (from not at all to strongly agree), that assess adolescents’ attitudes towards formal authority figures and institutions (teachers and police), and towards the transgression of school rules and social norms. The first factor, with five items, measures positive attitudes towards institutional authority (e.g., ‘I agree with what most teachers do and say’), and the second factor, with four items, assesses positive attitudes towards transgression of social norms (e.g., ‘If you don’t like a school rule, it’s best to break it’). The CFA using the maximum likelihood estimation method confirmed the fit of the proposed measurement model, SBχ2 = 66.59, df = 23, *p* < 0.001, CFI = 0.94, NNFI = 0.92, RMSEA = 0.05. Cronbach’s alpha coefficients in the present study were 0.88 and 0.85 for the dimensions of attitudes towards authority and attitudes towards transgression, respectively.

#### 2.2.4. School Violence

The Overt School Violence Scale [51] was used to assess overt school violence among peers. This scale has 13 items and a response scale ranging from 1—strongly disagree, to 4—strongly agree, and it measures overt violent behaviors towards peers in the school context. This scale measures general violence (e.g., “I am a person who hits others”), reactive violence (e.g., “When someone hurts me, I hit them”), and instrumental violence (e.g., “I threaten others to get what I want”). The CFA using the maximum likelihood estimation method confirmed the fit of the proposed measurement model, SBχ2 = 209.17, df = 65, *p* < 0.001, CFI = 0.91, NNFI = 0.91, RMSEA = 0.03. Cronbach’s alpha coefficient in this study was 0.90.

### 2.3. Procedure

First, a letter was sent to the principals of the selected schools to introduce the purpose of the project and ask for their participation. Seven schools voluntarily decided to participate. Second, a seminar was held for the teachers and parents from the selected schools to explain the research objectives and request parental permission. Third, a letter describing the study was sent to the parents, asking for their active consent for their child to participate in the study (96% of the parents authorized their children’s participation in the study). The scales were administered during a regular class period (55 min) with the supervision of trained researchers. Participation was voluntary and anonymous, and privacy was guaranteed. Few students refused to participate in the study (<2%). The same procedure was followed in both samples. This study was approved by the Ethics Committee of the University of Valencia (Protocol Number: H1456762885511). The imputation algorithm was employed to treat missing values, using the expectation-maximization method in both samples.

### 2.4. Data Analyses

First, Exploratory Factor Analysis (EFA) was performed with Sample 1, and the factors’ reliabilities were calculated. Bartlett’s sphericity test and the Kaiser–Mayer–Olkin (KMO) test were computed to assess the adequacy of the data for EFA. The Principal Components method with oblique rotation (Promax) was conducted to estimate the number of factors in cyberbullying perpetration. This rotation is used when the factors obtained are estimated to be correlated. To select the factors, an eigenvalue above 1 and a decrease in the Cattell sedimentation graphic were taken into account. All these analyses were made using SPSS program- version 26.

Next, Confirmatory Factor Analysis (CFA) was performed with Sample 2 to examine the factor structure yielded by the EFA. This procedure allowed us to obtain cross-validation of the scale’s factor structure [52,53]. The EQS 6.1 program was used [54]. The CFA model was estimated using maximum robust verisimilitude estimation, due to the lack of multivariate normality in the data (Mardia coefficient = 1573.56). Several indices were used to assess model fit, in addition to the Satorra–Bentler chi-square: the comparative fit index (CFI), the Bentler–Bonett non-normative fit index (NNFI)—also called the Tucker–Lewis index (TLI)—and the Root Mean Square Error of Approximation (RMSEA). CFI and TLI values above 0.95 indicate an adequate fit, as well as RMSEA values below 0.50. Regarding the Chi-square, a proposed model is considered to fit the data well when the ratio between the Chi-square and the degrees of freedom is less than three [55].

## 3. Results

### 3.1. Descriptive Findings: Sample 1

Table 1 reports the distribution of item responses, means, standard deviations, asymmetry, kurtosis, and item-total correlations for the CIB–AGS items. The most frequent behavior carried out by the participants was Item 2 (“I have called someone on the cellphone and hung up on him/her to bother or frighten him/her”), whereas the less frequent behavior was Item 3 (“I have threatened someone to make him/her do things on the Internet or smartphone that he/she did not want to do (like recording him/herself on video, giving me money, doing bad things”.). Kolmogorov–Smirnoff normality test results indicated that none of the items had a normal distribution. The results of the asymmetry and kurtosis tests indicated that all the items were skewed in a positive and leptokurtic way. Finally, the item-total correlations showed that all the items correlated positively and in the same direction.

### 3.2. Factor Structure of the CYB-AGS: Sample 1 and Sample 2

First, EFA was performed to examine the factors underlying the item set using the data from Sample 1. Bartlett’s sphericity test (χ^2^ = 11981.90; gl = 153, *p* < 0.001) indicated that the correlation matrix was factorable. KMO = 0.93 showed that the items had adequate common variance for EFA. Principal components analysis with oblique rotation (Promax) yielded a two-factor solution that accounted for 53.11% of the variance (see Table 2). The first factor accounted for 45.11% of the variance and included eight items referring to *indirect cyber-aggression*, which includes behaviors that are used by cyber-aggressors to avoid being identified and involve greater premeditation. The second factor accounted for 8.01% of the explained variance and included eight items that refer to direct cyber-bullying behaviors through electronic communication towards the victim that provoke an immediate negative effect. This factor has been called *direct cyber-aggression*. These categories are in line with the typology proposed by other authors [56,57,58,59]. The correlation between the two factors is high and significant (*r* = 0.65, *p* < 0.001).

CFA was conducted in the second sample (see Table 2) to examine the internal structure of the CYB-AGS. The tested model showed a good fit and confirmed the scale’s factor structure (χ^2^ = 189.79, df = 132, *p* < 0.001, CFI = 0.93, TLI = 0.92; RMSEA = 0.02 [0.01–0.02]). The internal consistency coefficients (Cronbach’s α) for these factors in the second sample was also above 0.70. Furthermore, high and significant correlations were obtained between the two factors (see Table 3).

### 3.3. Configural Invariance

After testing the measurement model, the invariance of the structure was tested. For this purpose, a multi-group analysis was carried out to confirm that the number of factors, the items’ saturations, and the correlations between the items were invariant in the two adolescent samples. Two nested models were estimated: (1) an unrestricted model in which there was no imposed equality between the saturations and the correlations between the factors (model without restrictions); and (2) a restricted model in which equality between all the saturations and the correlations between the factors (model with restrictions) in the groups were fixed. These models were compared using the χ^2^ difference test (See Table 4). The results showed that the two models did not differ when there were no restrictions; thus, the two models were equivalent.

### 3.4. Convergent Validity

Finally, to show convergent validity, correlations were examined among the two factors obtained with the CYB-AGS scale, the general index of Cyber-aggression, and other theoretically related constructs, such as: negative attitudes towards school, anger, attitudes towards institutional authority, attitudes towards transgression, and school violence (see Table 5).

The results showed that, on the one hand, indirect cyber-aggression, direct cyber-aggression, and the general index of cyber-aggression were positively correlated with negative attitudes towards school (r = 0.29, *p* < 0.001, r = 0.35, *p* < 0.001, and r = 0.35, *p* < 0.001, respectively), anger (r = 0.26, *p* < 0.001, r = 0.32, *p* < 0.001, and r = 0.33, *p* < 0.001, respectively), attitudes towards transgressions (r = 0.28, *p* < 0.001, r = 0.31, *p* < 0.001, and r = 0.33, *p* < 0.001, respectively), and school violence (r = 0.42, *p* < 0.001, r = 0.50, *p* < 0.001, and r = 0.51, *p* < 0.001, respectively). On the other hand, indirect cyber-aggression, direct cyber-aggression, and the general index of cyber-aggression correlated negatively with attitudes towards authority (r = 0.28, *p* < 0.001, r = 0.31, *p* < 0.001, and r = 0.33, *p* < 0.001, respectively).

## 4. Discussion

The increase in cyberbullying among adolescents is a serious worldwide social concern [13,60]. Technological changes lead to new cyberbullying behaviors that must be analyzed and adequately measured in the scientific field using updated and reliable instruments [5]. Thus, the main goal of the present study was to analyze the psychometric properties of the revised version of the Cyber-aggressor Scale. In this study, empirical evidence was found that confirms the validation of the CYB-AGS scale, its factor structure, its internal consistency, and its convergent validity with theoretically-related external variables.

Therefore, with regard to the factor structure of the CYB-AGS scale, the results of the present study suggest a two-factor structure. These factors, called direct cyber-aggressions and indirect cyber-aggressions, explain 53.12% of the variance. The first factor includes cyber-aggressions directed at the victim, verbal attacks (i.e., mocking someone in social networks), and social attacks (i.e., removing someone from groups to isolate them). The second factor consists of indirect or instrumental cyber-attacks that involve practices that cause indirect harm to the victim, such as creating a false profile of the victim, impersonating him or her, or hacking into his/her personal accounts.

These results are consistent with the two-dimensional structure of the CBVEQ-G scale obtained by Antoniadou et al. (2016) [45], and the two-factor structure of the CYB-VICS scale obtained by Buelga et al. (2019) [5] who classify the items into indirect aggressions and direct aggressions. In addition, these two dimensions of the CYB-AGS scale coincide with the suggestions of Langos (2012) [56] and Sarna et al. (2017) [58], who distinguish between direct and indirect cyberbullying to refer to private and public cyber-aggressions. For these authors, direct cyberbullying involves personal or private electronic communications, such as messages sent directly to the victim’s mobile phone. Indirect cyberbullying, rather than involving direct communication with the victim, is carried out indirectly over the Internet, for example, on Instagram when a photo of the victim is uploaded without his/her consent. The differentiation between direct and indirect cyberbullying is also consistent with the previous literature on traditional bullying, where some authors have demonstrated the existence of two dimensions of peer violence related to overt or direct aggression and relational or indirect aggression, respectively [5,51,61,62].

In addition, confirmatory factor analysis showed that the two-factor structure of the CYB-AGS scale was satisfactory, indicating a good fit of the model to the data. The invariance of the factor structure of the CYB-AGS scale was also confirmed with two independent samples of adolescents. Regarding the scale’s reliability, the results revealed an adequate reliability of the items by factors and on the total scale. Likewise, the analysis of the convergent validity of the scale showed significant correlations between the CYB-AGS scale and the external variables considered: aggressive behaviors in school, anger expression, negative attitude towards school, and transgression of norms.

Regarding the relationships between these variables, our results show interesting results that confirm the conclusions of previous studies on risk factors linked to cyberbullying. First, with the CYB-AGS scale, we confirmed the relationship between cyberbullying and violent behavior at school, which seems to support the idea suggested in the literature of an overlap between these two types of bullying behaviors towards peers [4,35,46,63]. In this direction, Lee and Shin (2017) [64] and Ortega-Baron et al. (2017) [46] argue that being a traditional or face-to-face bully is a predictor of cyberbullying. Giumetti and Kowalski (2016) [63] indicate that 88% of victims and bullies at school play the same role in the online environment. In addition to supporting the continuity of the aggressor’s role in different environments, this result also indicates that the perpetrator is involved in a constellation of other antisocial and maladaptive behaviors, as many authors have suggested [43,60].

Certainly, as observed in this study, adolescents’ attitudes towards social order and norms are associated with their social behavior. Thus, we found, as expected, that a positive attitude towards the transgression of social norms is related to the adolescent’s involvement in cyberbullying behavior, whereas a positive attitude towards institutional authority (police, teachers) is negatively related to cyberbullying. This result supports the large amount of research showing the importance of negative attitudes towards authority as a risk factor for adolescent involvement in a variety of antisocial behaviors [46,65,66,67]. Along these lines, adolescent offenders with antisocial behaviors have also been found to present significant school problems related to poor school performance and school rejection [20,22,66]. In our study, this idea is corroborated by the significant link found between adolescent involvement in cyberbullying behavior and negative attitudes towards school. Likewise, anger is another variable in our study that was associated with cyberbullying [63]. In the research by Gradinger, Strohmeier and Spiel (2010) [68], the authors found that anger is the first reason or motive cyberbullies attribute to their bullying behavior. Thus, a large number of authors have found, as we did, that cyberbullying is related to anger control issues [69,70,71].

The present study has some limitations. Although the sample size used in this research is adequate for the analyses carried out [53,72], future research should extend the sample, both nationally and internationally. By using international samples, the existence of possible cultural differences in the measurement of the instrument could be taken into account. In addition, the scale could be validated not only in urban populations, as in this study, but also in rural populations. The conditions and lifestyles of adolescents in rural populations are different from those living in urban settings. Finally, another study limitation is that, although evidence is provided about relevant issues related to the psychometric properties of the instrument (factorial validity, convergent validity, and reliability), other psychometric issues such as test–retest reliability are not included. It would be interesting to include these analyses in future research.

Nevertheless, and despite these limitations, the present study confirms the suitability of the CYB-AGS scale based on its adequate psychometric properties, which makes its application in adolescent populations highly recommended. In an increasingly technological and hyper-connected society like ours, validated instruments such as the CYB-AGS scale are necessary in order to measure and evaluate new forms of cyberbullying with scientific rigor and develop programs to prevent this growing global problem in adolescents and increasingly younger children.

## 5. Conclusions

Since cyberbullying emerged as a public health problem, the construct of cyberbullying has evolved to include new behaviors. As a consequence, there is a need to provide new and updated measurement tools that address the range of intimidation through the Internet. This study provides empirical evidence about the validation of the CYB-AGS scale, its factor structure, its internal consistency, and its convergent validity with theoretically-related variables. The results of the present study yielded a two-factor structure that assess both direct and indirect cyber-aggressions. Direct cyber-aggressions are behaviors and attacks directed at another person, both verbal and social. Indirect cyber-aggressions are behaviors and attacks that use content manipulation, identity theft and hacking. CFA demonstrated that the two-factor structure of the CYB-AGS scale was satisfactory, indicating a good fit of the model to the data. The invariance of the factor structure of the CYB-AGS scale was also observed with two independent samples of adolescents. The analysis of the convergent validity of the scale revealed significant correlations between the CYB-AGS scale and aggressive behaviors in school, anger expression, negative attitude towards school, and transgression of norms.

This scale might be a useful tool for teachers, psychologists, and head principals to provide useful information about the prevalence of cyber perpetrators at school and therefore to develop preventive programs to minimize cyberbullying. 

## Figures and Tables

**Table 1:** Descriptive statistics for the cyber aggressor scale (CYB-AGS).

	Frequency Distribution (%)					M	SD	Asym. (S.E. = 0.06)	Kurt. (S.E. = 0.13)	K-S	*r_i-t_*
Item	Never	Once or Twice	A Few Times (3–5)	Several Times (6–10)	Many Times (+10)						
CB1	980 (77.4)	289 (22)	31 (2.4)	8 (0.6)	10 (0.8)	1.22	0.57	3.79	17.35	0.39 ***	0.52
CB2	940 (71.3)	321 (24.4)	37 (2.8)	10 (0.8)	10 (0.8)	1.27	0.65	3.32	4.65	0.38 ***	0.50
CB3	1131 (85.8)	273 (23.1)	9 (7)	4 (0.3)	1 (0.1)	1.04	0.27	8.63	84.29	0.48 ***	0.67
CB4	1048 (79.5)	241 (18.3)	19 (1.4)	7 (0.5)	3 (0.2)	1.14	0.43	4.65	26.39	0.41 ***	0.58
CB5	1099 (83.4)	194 (14.7)	15 (1.1)	7 (0.5)	3 (0.2)	1.09	0.39	6.23	44.34	0.45 ***	0.58
CB6	1089 (82.6)	203 (15.4)	19 (1.4)	4 (0.3)	3 (0.2)	1.09	0.38	5.86	40.65	0.44 ***	0.57
CB7	1115 (84.6)	186 (14.1)	11 (0.8)	4 (0.3)	2 (0.2)	1.06	0.32	7.39	63.97	0.46 ***	0.73
CB8	1121 (85.1)	182 (13.8)	5 (0.4)	7 (0.5)	3 (0.2)	1.06	0.33	8.18	74.56	0.47 ***	0.69
CB9	1127 (85.5)	180 (13.6)	5 (0.4)	4 (0.3)	2 (0.2)	1.05	0.28	9.02	94.68	0.47 ***	0.69
CB10	1106 (83.9)	196 (14.8)	8 (0.6)	7 (0.5)	1 (0.1)	1.07	0.32	6.76	53.08	0.45 ***	0.64
CB11	973 (73.8)	299 (22.7)	32 (2.4)	9 (0.7)	5 (0.4)	1.22	0.53	3.51	15.15	0.39 ***	0.56
CB12	1127 (85.5)	170 (12.9)	11 (0.8)	9 (0.7)	1 (0.1)	1.06	0.33	7.53	60.49	0.47 ***	0.64
CB13	1005 (76.3)	259 (19.7)	30 (2.3)	12 (0.9)	12 (0.9)	1.21	0.59	3.98	18.02	0.40 ***	0.59
CB14	1049 (79.6)	233 (17.6)	25 (1.9)	8 (0.6)	3 (0.2)	1.14	0.45	4.53	24.14	0.41 ***	0.67
CB15	1056 (80.1)	232 (17.6)	16 (1.2)	10 (0.8)	4 (0.3)	1.13	0.45	5.02	29.57	0.46 ***	0.67
CB16	1110 (84.2)	194 (14.8)	7 (0.5)	1 (0.1)	5 (0.4)	1.07	0.35	8.19	78.41	0.46 ***	0.59
CB17	1101 (83.5)	201 (15.2)	7 (0.5)	3 (0.2)	6 (0.5)	1.08	0.37	7.47	65.05	0.45 ***	0.53
CB18	1022 (77.5)	261 (19.8)	7 (0.5)	3 (0.2)	6 (0.5)	1.15	0.45	4.15	21.23	0.41 ***	0.65

Note: M = mean; SD = standard deviation; Asym. = asymmetry; Kurt. = kurtosis; SE = standard error; K-S = Kolmogorov–Smirnoff; ri-t = corrected item–total correlation. *** *p* < 0.001.

**Table 2:** Factor loadings for the EFA and CFA.

Item	EFA		CFA	
	F1	F2	F1 ICB	F2 DCB
CB1	0.39	0.69	0.66	
CB2	0.39	0.64	0.50	
CB3	0.81	0.49		0.71
CB4	0.54	0.62	0.68	
CB5	0.65	0.49		0.69
CB6	0.67	0.43		0.63
CB7	0.84	0.57		0.67
CB8	0.83	0.51		0.75
CB9	0.78	0.55		0.72
CB10	0.74	0.49		0.69
CB11	0.42	0.73	0.75	
CB12	0.77	0.46		0.69
CB13	0.48	0.70	0.73	
CB14	0.56	0.75	0.77	
CB15	0.55	0.68	0.73	
CB16	0.67	0.50		0.70
CB17	0.64	0.41		0.65
CB18	0.51	0.78	0.77	
% Explained Variance	45.11	8.01		

Note: ICB = Indirect Cyber-aggression; DCB = Direct Cyber-aggression; EFA = Exploratory Factor Analysis; CFA = Confirmatory Factor Analysis.

**Table 3:** Descriptive statistics, factor correlations, and Cronbach’s alpha (in parentheses) for the two-factor model.

	M	SD	DCB	ICB
DCB	1.07	0.35	(0.86)	
ICB	1.18	0.52	0.67 **	(0.93)

Note: DCB = Direct Cyber-aggression, ICB = Indirect Cyber-aggression, M = mean; SD = Standard Deviation. Alpha Global Scale = 0.94, ** *p* < 0.01.

**Table 4:** Tests of measurement invariance across two samples for the two-factor model.

Model	Description	χ^2^	df	Comparison of Nested Models	Difference S-B χ^2^	Difference df	*p*
M1	Restricted model	360.1701	284		-	-	-
M2	Model without restrictions	360.0521	268	M1–M2	0.118	16	>0.05

**Table 5:** Correlations among cyber-aggression, negative attitude towards school, attitude towards institutional authority, attitude towards transgressions, and overt school violence.

	NAS	Anger	AA	AT	OvSV
Indir. Cyber-aggression	0.29 ***	0.26 ***	−0.20 ***	0.28 ***	0.42 ***
Direct Cyber-aggression	0.35 ***	0.32 ***	−0.26 ***	0.31 ***	0.50 ***
Total Cyber-aggression	0.35 ***	0.33 ***	−0.26 ***	0.33 ***	0.51 ***

Note: NAS = Negative Attitude Towards School; AA = Attitude towards institutional Authority; AT = Attitude towards Transgressions; OvSV = Overt School Violence. *** *p* < 0.001.

## Appendix A. CYBAGS

**Adolescent Cyber-Bullying Scale**

Below, you will see some behaviors that some boys and girls might do to intimidate or really bother you (not as a joke) through the cellphone, Internet, social networks, tablets, or WhatsApp in the past year.

**1: Never**	**2: Once or twice**	**3: A few times** (between 3 and 5)	**4: Several times** (between 6 and 10)	**5. Many times** (more than 10)
1. I have insulted or ridiculed someone in social networks or groups like WhatsApp to really screw with or annoy him/her. [He insultado o puesto en ridículo en redes sociales o en grupos como el WhatsApp para que se fastidie o se moleste de verdad]				1 2 3 4 5
2. I have called someone’s cellphone and hung up to bother or frighten him/her. [He llamado al móvil y no he contestado para que se fastidie o se asuste]				1 2 3 4 5
3. I have threatened someone to make him/her do things on the Internet or smartphone that he/she did not want to do (like recording him/herself on video, giving me money, doing bad things). [He obligado con amenazas a hacer cosas que no quería en Internet o por el móvil (como grabarse en video, darme dinero, hacer cosas malas)]				1 2 3 4 5
4. I have told someone’s secrets or revealed personal things about him/her in social networks or groups (WhatsApp, snapchat…). [He contado secretos o revelado cosas personales de alguien sin su permiso en redes sociales o en grupos (whatsapp, snapchat..)]				1 2 3 4 5
5. To make fun of someone, I have made or manipulated videos or photos of him/her and uploaded or distributed them on social networks or by smartphone. [Para burlarme de alguien, he creado o manipulado videos o fotos mías, y las he subido o distribuido en redes sociales o por el móvil].				1 2 3 4 5
6. I’ve logged into someone’s profile or accounts, and he/she could not do anything about it. [He entrado en el perfil o en cuentas de una persona sin que ella pueda hacer nada]				1 2 3 4 5
7. I have pretended to be someone else so I could say or do bad things on the Internet. [Me he hecho pasar por otra persona para decir o hacer cosas malas en Internet]				1 2 3 4 5
8. I have purposely created a webpage, a forum, or a group just to make fun of someone and criticize him/her in front of everyone. [He creado adrede una página, un foro o un grupo solo para meterme con una persona y criticarle delante de todos]				1 2 3 4 5
9. I have put someone’s cellphone number on the Internet and said bad or false things about him/her so that people would call him/her and get him/her into trouble. [He puesto el número de teléfono móvil de alguien en Internet diciendo cosas malas o falsas de esa persona para que la llamen y meterla en líos]				1 2 3 4 5
10. I have taken someone’s smartphone and used it to send photos, videos, or mean messages to others to get him/her into trouble with them. [He cogido el teléfono de alguien y desde su móvil he enviado fotos, videos o mensajes malos a otros para meterla en problemas]				1 2 3 4 5
11. I have criticized someone or made fun of comments, photos, or videos he/she uploaded to social networks or groups like WhatsApp. [He criticado o me he burlado de comentarios, fotos o videos que una persona ha subido en redes sociales o en grupos como el WhatsApp]				1 2 3 4 5
12. I have created a false profile on the Internet with someone’s personal data in order to impersonate him/her saying or doing bad things. [He creado en Internet un perfil falso con datos personales de alguien para decir o hacer cosas malas, haciéndome pasar por él/ella]				1 2 3 4 5
13. I have ignored and did not answer someone’s messages or things he/she shared in groups or social networks, just to make him/her feel bad. [He ignorado y no he contestado a mensajes o cosas que alguien ha puesto en grupos o en redes sociales para hacerle sentir mal]				1 2 3 4 5
14. I have provoked someone in social networks or groups by insulting or taunting him/her to make him/her angry and cause a big argument. [He provocado a alguien en redes sociales o en grupos con insultos y burlas para que se enfade mucho y haya una gran discusión]				1 2 3 4 5
15. I have eliminated or blocked someone from groups to leave him/her without any friends. [He eliminado o bloqueado a alguien de grupos para dejarle sin amigos]				1 2 3 4 5
16. I’ve stolen photos, videos, or private conversations and uploaded them or sent them to others. [He robado fotos, videos, conversaciones privadas y las he subido o enviado a otros]				1 2 3 4 5
17. I have changed someone’s password to social networks so that he/she could not access them. [He cambiado la contraseña de las redes sociales de una persona para que no pueda entrar en ellas]				1 2 3 4 5
18. I sent someone tauntin messages to bother and annoy him/she. [He enviado a alguien mensajes de burla para fastidiarle y molestarle a otros]				1 2 3 4 5
